# Contribution to Orthopedic Surgery

**Published:** 1899-05

**Authors:** 


CONTRIBUTION TO ORTHOPEDIC SURGERY. By A. SYDNEY ROBERTS, M. D., late Surgeon to the Philadelphia Hospital; Orthopedic Surgeon to the Out-Patient Department in the University Hospital; Instructor in Orthopedic Surgery in the University of Pennsylvania; Out-Patient Surgeon to the Episcopal Hospital; Fellow of the College of Physicians of Philadelphia and of the American Orthopedic Association; Member of the Pennsylvania State Medical Society, Philadelphia County Society, Pathological Society, Neurological Society, etc., With a brief Biographical Sketch, by James K. Young, M. D., Professor of Orthopedic Surgery, Philadelphia Polyclinic; Professor of Orthopedic Surgery, Woman's Medical College, etc. Illustrated, pp. 300 Philadelphia: William J. Dornan, Printer. 1898. From the Editor.

This volume is a rare jewel in more ways than one. It is a splendid contribution to orthopedics, a grouping of masterly, clear cut gems; it is a monument erected by a devoted friend to the memory of one whose work has been cut short by death In this latter respect it is a mark of friendship and gratitude too infrequently shown.

In his preface Dr. Young says: "The present collection is undertaken for private distribution only, for the sole purpose of increasing the interest in the subjects of which it treats, and is prepared by the editor as a debt of gratitude for many past kindnesses."

The introduction written with a deep sense of gratitude and reverence by Dr. Young is a fitting bit of work for such a collection. Dr. Young calls attention to the custom among the members of the bar of holding a general bar meeting, presided over by a judge to pay tributes to a dead member, and regrets that no such custom obtains in the medical profession.

"In lieu of such a custom," writes Dr. Young, "it seems highly fitting that I. as pupil, friend, successor, upon whom in some measure his mantle has fallen, should pay my tribute to the sacred memory of the departed. When one of the medical profession whose ability and attainments mark his individuality preëminent passes into eternity, those who linger make a note of his scholarly attainments and professional skill and emulate his many virtues."

A sketch of Dr. Roberts's life is given in narrative form. It tells of his studies with Dr. W. W. Keen and of his graduation in 1877 in a class which numbered among its members James M. Anders, Joseph Price, Thomas H. Fenton, Rush D. Huidekoper, William Hobson Heath, of Buffalo, who is now professor of genito-urinary diseases in the Medical Department of the University of Buffalo; John H. Musser, Andrew J. Parker and George A. Piersoll.

Dr. Roberts's labors in orthopedics were begun at the suggestion of Dr. S. Weir-Mitchell. It was through his personal efforts that the orthopedic shop in the University Hospital was organized. The continuation of this shop and the furnishing of free apparatus to the poor is secured by the A. Sydney Roberts Apparatus Fund, endowed by Dr. Roberts's family.

From this point on Dr. Young pays tribute to Dr. Roberts in graceful language and tells of his many manly qualities and his deep and lasting friendships. Reference to his professional abilities is made without gush, without slathering praise. His work is spoken of in earnest admiration and his death is referred to with touching simplicity.

The works of Dr. Roberts which are printed are very complete and most valuable as contributions to surgical literature. They are : Club-foot: Talipes; Potts's Disease; The Spinal Arthropathies; Clinical Lectures on Orthopedic Surgery; Flat-foot: A new plantar spring for its relief; Chronic Articular Osteitis of the Knee-joint and description of a new mechanical splint; Deformity of the Fore-arm and Hands.

Dr. Young has paid a graceful tribute to a dead friend and has made in his introductory remarks a suggestion which is worthy of adoption : that the medical profession should follow the example of the legal profession and publicly pay tribute to dead fellow-workers.

